# The Double-Edged Sword: Legumain as a Context-Dependent Mediator and Therapeutic Target in Cancer and Cardiovascular Disease

**DOI:** 10.3390/cells15181678

**Published:** 2026-09-16

**Authors:** Siarhei A. Dabravolski, Gulalek A. Babayeva, Daria D. Borodko, Ulyana V. Rozhkova, Stanislav A. Antonov, Aleksandra S. Utkina, Maria O. Nerush

**Affiliations:** 1 Department of Biotechnology Engineering, Braude Academic College of Engineering, Snunit 51, P.O. Box 78, Karmiel 2161002, Israel; sergedobrowolski@gmail.com; 2 Smirnov Institute of Experimental Cardiology, Chazov National Medical Research Center of Cardiology, 15-A, Academician Chazov Street, 121552 Moscow, Russia; 3 Laboratory of Molecular Genetic Modeling of Inflamaging, Institute of General Pathology and Pathophysiology, 8, Baltiiskaya Street, 125315 Moscow, Russia; 4 Institute for Atherosclerosis Research, 4-1-207, Osennyaya Street, 121609 Moscow, Russia; 5 Scientific and Educational Center for Molecular and Cellular Technologies, St. Petersburg Chemical Federal University, 21 Rentgen Street, 197101 St. Petersburg, Russia

**Keywords:** legumain, asparaginyl endopeptidase, cysteine protease, cancer, cardiovascular disease, tumour microenvironment, atherosclerosis, therapeutic target, biomarker, inflammation

## Abstract

Legumain, an asparaginyl endopeptidase, has emerged as a critical cysteine protease with diverse and complex roles in human health and disease. While traditionally known for its function in endolysosomal protein degradation, a growing body of evidence has revealed its presence in multiple cellular compartments and its involvement in a vast array of pathological processes. This review provides a comprehensive synthesis of the current literature, bridging the often-siloed research in oncology and cardiovascular medicine to present a unified view of legumain’s function. We first detail the multifaceted role of legumain in cancer, where it acts as a driver of tumour proliferation, invasion, and metastasis. We explore its function as a modulator of the tumour microenvironment, particularly its ability to polarise macrophages towards an immunosuppressive phenotype, and discuss its emerging value as a candidate prognostic biomarker and a versatile therapeutic target. Notably, however, therapeutic targeting of legumain remains predominantly at the preclinical stage. Subsequently, we examine its significant contribution to cardiovascular diseases, detailing its mechanistic involvement in promoting atherosclerosis, hypertension, and adverse cardiac remodelling through pathways involving inflammation, extracellular matrix turnover, and immune cell modulation. By synthesising these findings, we highlight the convergent pathological mechanisms, discuss current controversies and methodological limitations—such as the lack of standardised assays—and propose key future directions. This review frames legumain as a biological rheostat and a potentially important node in chronic disease, underscoring its potential as a powerful biomarker and a sophisticated therapeutic target across a broad spectrum of human pathologies.

## 1. Introduction

Proteases are essential regulators of nearly all physiological processes, from protein turnover and cell signalling to tissue remodelling and immune responses. Within this vital class of enzymes, the cysteine proteases play a particularly crucial role in cellular homeostasis and disease. Among them, legumain stands out as a unique and functionally complex member. First discovered in leguminous plants [1], its mammalian counterpart, asparaginyl endopeptidase (AEP) or δ-secretase, was later identified and characterised as a key endolysosomal cysteine protease encoded by the *LGMN* gene [2]. For clarity, we use “legumain” as the general term for the protein; “AEP” only where it reflects the terminology of the cited primary source; “prolegumain” or “zymogen” for the inactive 56 kDa precursor; “mature” or “active legumain” for the processed, catalytically competent forms; and italicised “LGMN” exclusively for the gene or its transcript/pseudogene products. Although highly expressed in tissues such as the kidneys, liver, and spleen, its significance extends far beyond basal cellular functions, positioning it at the crossroads of health and pathology [3].

The biological activity of legumain is governed by a sophisticated, multi-layered regulatory system. Synthesised as an inactive 56 kDa zymogen, prolegumain undergoes post-translational modifications, including N-glycosylation and ubiquitination, which are critical for its transport, stability, and localisation [4]. Within the acidic environment of the endolysosome, it undergoes autocatalytic processing to yield its mature, active forms [5]. This pH-dependent activation mechanism confers upon legumain a remarkable functional plasticity. Depending on the precise pH and local environment, it can exhibit distinct enzymatic activities: classical AEP activity at mildly acidic pH (~5.8), asparaginyl-specific carboxypeptidase (ACP) activity at near-neutral pH, and even caspase-like aspartyl-peptidase activity under highly acidic conditions (<4.0) [6,7]. Furthermore, under specific conditions, legumain functions as a peptide asparaginyl ligase (PAL), capable of catalysing peptide bond formation, a rare activity for a protease [8,9]. This intricate regulation is further modulated by structural elements, such as the legumain stabilisation and activity-modulating (LSAM) domain, and by endogenous inhibitors like cystatins C, E/M, and F, which finely tune its proteolytic potential [10,11].

While traditionally considered an endolysosomal protease, a growing body of evidence has revealed legumain’s presence in multiple cellular and extracellular compartments, including the cytosol, nucleus, cell surface, and in secreted forms within the secretome and extracellular vesicles [12,13,14,15]. This widespread distribution points to a functional repertoire far exceeding simple protein degradation. Extracellular legumain, for instance, participates in remodelling the pericellular environment: it activates matrix metalloproteinases such as MMP-2, a mechanism detailed in the cancer and cardiovascular contexts below (Section 2.2 and Section 3.1) [16], and it regulates the lysosomal turnover of the cell-surface metalloproteinases ADAM10 and ADAM17 [17]. On the cell surface, its interaction with integrins can further modulate signalling cascades, while its presence in the nucleus suggests a role in regulating transcription [18,19]. This subcellular diversity underscores legumain’s capacity to influence a vast array of biological processes.

Consequently, the dysregulation of legumain expression and activity has emerged as a central feature in a wide spectrum of human diseases. Its overexpression is strongly implicated in the progression, invasion, and angiogenesis of numerous cancers, including colorectal, breast, and gastric malignancies, making it a prominent biomarker and therapeutic target in oncology [20,21]. Similarly, legumain contributes to the pathogenesis of cardiovascular diseases by promoting atherosclerosis, aortic aneurysm, and pulmonary hypertension [22]. Its role extends to fibrosis, neurodegenerative diseases such as Alzheimer’s disease and Parkinson’s disease, and processes associated with ageing and senescence [16,23,24,25,26]. While research within these individual fields has flourished, the findings often remain siloed. A comprehensive review that bridges the fundamental biochemistry and cell biology of legumain with its multifaceted roles across these diverse disease contexts is therefore timely and necessary.

This review addresses this gap by providing a comprehensive and critical synthesis of the literature. As will become clear, one of the most consistent findings to emerge from this synthesis is that legumain’s biological impact is highly context-dependent—varying with cell type, disease stage, and the acute-versus-chronic setting—rather than reflecting a single, universally pathogenic mechanism. We propose that this context-dependent functional plasticity, rather than a unifying “master regulator” role, is the most useful lens through which to interpret the evidence presented below. We will begin by exploring the multifaceted role of legumain in oncology (Section 2), examining its function as a prognostic biomarker, a mechanistic driver of tumour progression, a modulator of the tumour microenvironment, and a versatile therapeutic target. Next, we will shift our focus to cardiovascular disease (Section 3), detailing legumain’s contribution to atherosclerosis, hypertension, and adverse cardiac remodelling, while evaluating its utility as a clinical biomarker. Subsequently, in Section 4, we will synthesise these disparate findings, discussing the convergent pathological pathways, unresolved controversies, and current methodological limitations. Finally, this review will conclude (Section 5) by proposing key future research directions aimed at bridging the existing knowledge gaps and translating our understanding of this remarkable protease into clinical benefit.

### Literature Search Strategy

As a narrative review, this article does not follow a formal systematic-review protocol; nevertheless, a structured search was conducted to ensure comprehensive coverage. The PubMed/MEDLINE, Scopus, and Web of Science databases were searched for articles published between ч and 05.26, using combinations of the terms “legumain”, “asparaginyl endopeptidase”, “AEP”, “LGMN”, “cancer”, “tumour”, “cardiovascular disease”, “atherosclerosis”, and related terms (e.g., specific cancer types, “biomarker”, “therapeutic target”). Only peer-reviewed, English-language articles reporting in vitro, in vivo, or clinical/human data on legumain biology were considered. Reference lists of retrieved articles were additionally screened to identify further relevant publications. The final search was conducted in May 2026. Studies were included if they reported original in vitro, in vivo, or human clinical/observational data on legumain (asparaginyl endopeptidase) biology in the context of cancer or cardiovascular disease; reviews without primary data, non-English-language articles, and conference abstracts without accompanying full-text publication were excluded. Given the narrative, non-systematic nature of this review, publication bias and search-strategy limitations cannot be excluded.

## 2. The Role of Legumain in Cancer

The dysregulation of legumain is a hallmark of numerous malignancies, where it functions as an active driver of tumour progression and a candidate prognostic biomarker (Table 1 and Table 2). Following a logical progression from fundamental biology to clinical application, this section will first detail the molecular mechanisms governing legumain expression, then explore its functional roles in preclinical models of cancer and finally establish its clinical significance in human disease. This body of evidence provides the foundation for the concluding discussion on legumain as a promising therapeutic target.

### 2.1. Fundamental Regulation of Legumain Expression and Activity

**Figure 1:**
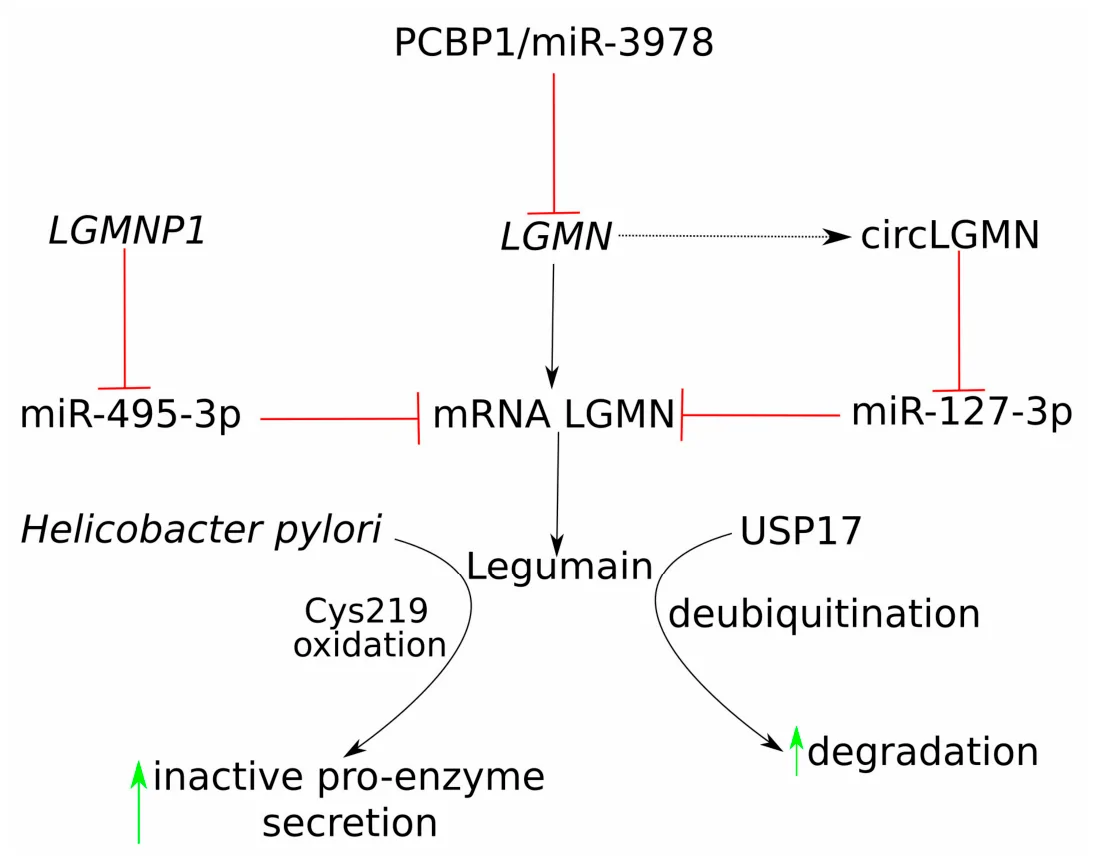
The Regulatory and Functional Network of Legumain in Cancer. Legumain pseudogene LGMNP1 sponges miR-495-3p, thus regulating LGMN expression and modulating cell proliferation, migration, and invasion. CircLGMN sponges miR-127-3p, thus regulating LGMN expression and modulating tumour cell function. RNA-binding protein PCBP1 forms a complex with miR-3978 to suppress legumain expression. *Helicobacter pylori* oxidase Cys219 in legumain, promoting secretion of the inactive pro-enzyme. USP17 directly deubiquitinates legumain, thereby targeting it for degradation.

At the most fundamental level, the oncogenic role of legumain is dictated by a complex network of regulatory molecules that control its expression and activity, which are often hijacked in cancer (Figure 1). Studies, primarily in cell culture models, have revealed that this control occurs at multiple levels.

In glioblastoma (GBM), legumain expression is controlled by a competing endogenous RNA (ceRNA) network. A functional pseudogene, LGMNP1, has been shown to act as a sponge for miR-495-3p, thereby preventing this microRNA from targeting and suppressing *LGMN* mRNA [41]. Similarly, a circular RNA derived from the *LGMN* gene itself, circLGMN, sequesters miR-127-3p, leading to increased legumain protein levels and promoting GBM malignancy [42].

Post-transcriptional and external factors also play a crucial role. In gastric cancer, the RNA-binding protein PCBP1 forms a complex with miR-3978, which appears to suppress legumain expression. During metastatic progression, this complex is disrupted, leading to legumain upregulation and chemoresistance [47]. Furthermore, infection with the bacterium *Helicobacter pylori* can induce oxidative modification of a key cysteine residue (Cys219) in legumain, inhibiting its intracellular processing and enhancing the secretion of the inactive pro-enzyme, thus establishing a unique link between infection, redox regulation, and tumorigenesis [48]. In breast cancer, the deubiquitinase USP17 directly interacts with and deubiquitinates legumain, targeting it for degradation and thereby inhibiting tumorigenesis through the inactivation of ERK signalling [33].

### 2.2. Functional Roles of Legumain in Preclinical Cancer Models

**Figure 2:**
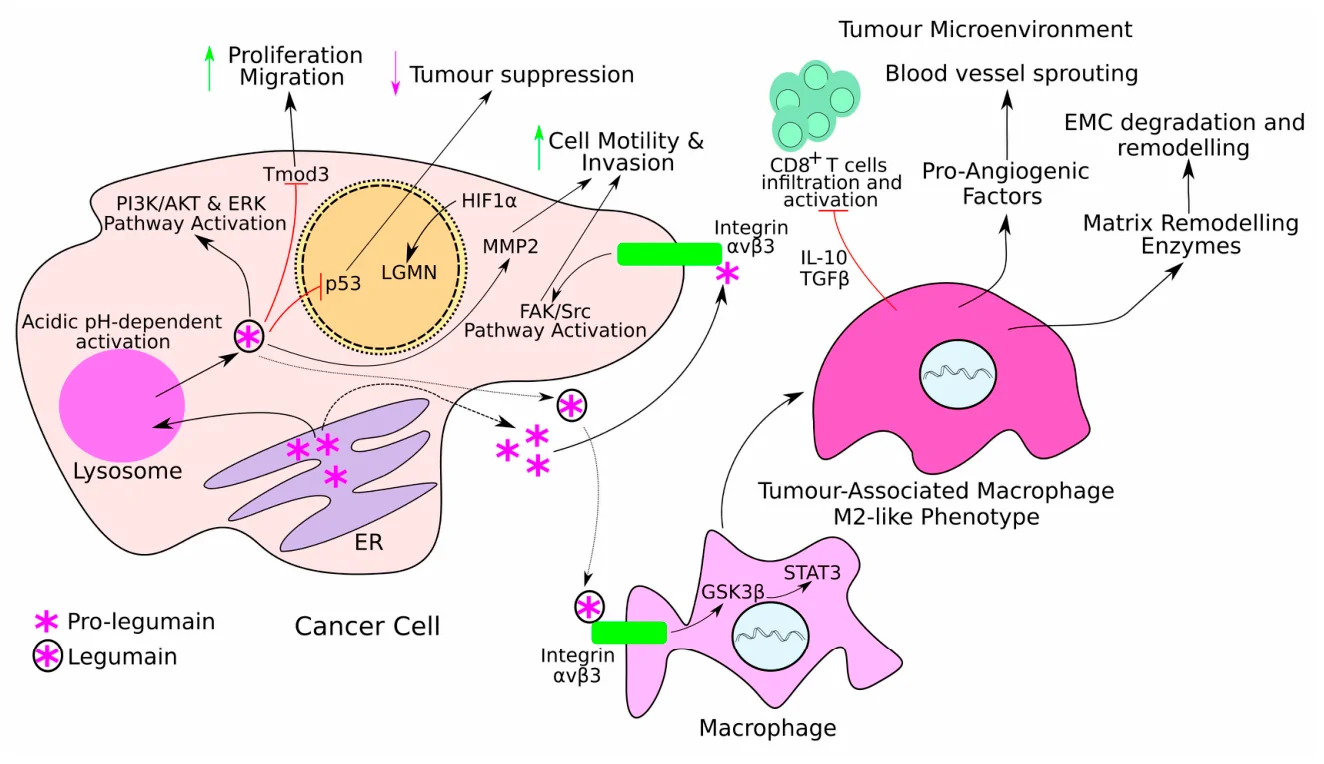
The Mechanistic Roles of Legumain as a Driver of Cancer Progression. Within the cancer cell, pro-legumain is processed to its active form in the acidic lysosome, where it cleaves substrates like p53 and Tmod3 to drive proliferation and migration. HIF1α transcriptionally regulates LGMN. Secreted pro-legumain can also act in an autocrine fashion, binding to integrin αvβ3 to activate pro-invasive signalling. Also, legumain activates MMP2, promoting metastasis. In the tumour microenvironment, legumain secreted by cancer cells acts in a paracrine manner on macrophages, promoting their polarisation to an immunosuppressive M2-like phenotype. These TAMs then remodel the TME by releasing factors that inhibit anti-tumour immunity and promote angiogenesis.

Building upon these fundamental mechanisms, in vivo studies using animal models have been instrumental in demonstrating legumain’s causal role in driving the hallmarks of cancer (Figure 2). These models confirm that the dysregulation observed in vitro translates into tangible, pro-tumoural functions in a complex biological system (Table 1).

Preclinical models consistently show that legumain promotes tumour growth and spread. In a subcutaneous mouse model of epithelial ovarian cancer (EOC), forced overexpression of legumain enhanced tumour growth and microvessel density [53]. Likewise, in choriocarcinoma xenografts, legumain overexpression was sufficient to reverse the anti-proliferative and anti-invasive effects of metformin [57].

Legumain’s pro-metastatic activity is a key feature in animal models. In mouse models of breast cancer, legumain promotes lung metastasis by cleaving and activating matrix metalloproteinase-2 (MMP2) [27] and by activating FAK-Src-RhoA signalling through a non-proteolytic interaction with integrin αvβ3 [28]. Furthermore, it has been shown to be crucial for activating the PI3K/AKT pathway, with elevated serum levels correlating with tumour progression in transgenic mouse models [30]. It also promotes epithelial–mesenchymal transition (EMT) by activating the ERK pathway via its regulation of CD74 [31] and directly regulates autophagy and lysosomal function by cleaving the p85 subunit of PI3K [32]. The C/EBPβ/AEP signalling pathway has also been shown to mediate oxidative stress and metastasis, which could be mitigated by a legumain inhibitor [35]. Its activity also contributes to the painful consequences of cancer; in a mouse model of breast cancer bone metastasis, legumain aggravated bone destruction and pain-like behaviours [29]. Similarly, in mouse models of oral squamous cell carcinoma (OSCC), legumain was shown to cause cancer-associated pain by activating PAR2 on trigeminal neurons [58]. In glioblastoma xenograft models, legumain promotes tumour progression by cleaving tropomodulin-3, which enhances both cell migration and proliferation [39]. In the context of colitis and the transition to colorectal cancer, proteomic analyses have identified potential direct and indirect substrates of legumain, shedding light on its function in the inflammatory tumour landscape [50].

Animal models have been critical in revealing legumain as a master regulator of the TME, creating an immunosuppressive niche that facilitates tumour growth. This is largely achieved by influencing immune cell populations, particularly macrophages. In mouse models of gastric cancer, legumain secreted by tumour cells was shown to polarise macrophages towards a pro-tumoural M2 phenotype and promote immune evasion [12], while legumain expressed by TAMs themselves stimulated cancer cell proliferation and angiogenesis [46]. This effect is central in glioblastoma mouse models, where TAM-derived legumain activates immunosuppressive signalling pathways and promotes both macrophage infiltration and GBM cell proliferation, thereby compromising anti-tumour immunity [44,45]. This axis can be driven by the tumour’s core circadian clock machinery, which upregulates legumain to control microglial infiltration and function [43]. In contrast, models of breast cancer have shown that legumain deletion in macrophages can induce tumour cell senescence, highlighting context-dependent functions [34]. In pancreatic cancer, exosomes from cancer cells were found to upregulate legumain in dendritic cells, suggesting a potential role in mediating immune escape [55].

### 2.3. Clinical Significance and Prognostic Value of Legumain in Human Cancers

The functional roles established in preclinical models are validated by extensive data from human studies, which firmly position legumain as a clinically relevant biomarker for prognosis, diagnosis, and therapy response (Table 2).

High legumain expression in patient tumour samples consistently correlates with poor outcomes. This has been demonstrated in ductal carcinoma in situ (DCIS), where it is an independent predictor of recurrence [59], as well as in thyroid carcinoma [54] and gastric cancer, where it is associated with poor overall survival and peritoneal metastasis [61]. Circulating legumain levels also hold diagnostic and prognostic power, as shown in glioblastoma, where plasma levels correlate with tumour size and decrease after surgery [40], and in EOC, where exosomal legumain is associated with metastasis and poor survival [19]. While its prognostic value is strong, it can be context-specific, as seen in B-cell precursor acute lymphoblastic leukaemia (BCP-ALL), where it was not significantly associated with relapse-free survival [62].

Furthermore, patient data link legumain to therapy resistance. In patients with metastatic colorectal cancer, higher legumain expression was associated with shorter progression-free survival on cetuximab therapy [49]. In breast cancer patients, high legumain levels were associated with radiotherapy resistance and correlated with the suppression of the DNA damage response pathway [60]. It should be noted that these clinical associations are based on measurements of total immunoreactive legumain rather than its enzymatic activity (see Section 4.3); whether the reported prognostic value reflects increased active enzyme, altered processing, or simply increased protein abundance remains to be determined.

### 2.4. Legumain as a Target and a Tool in Cancer Theranostics

**Figure 3:**
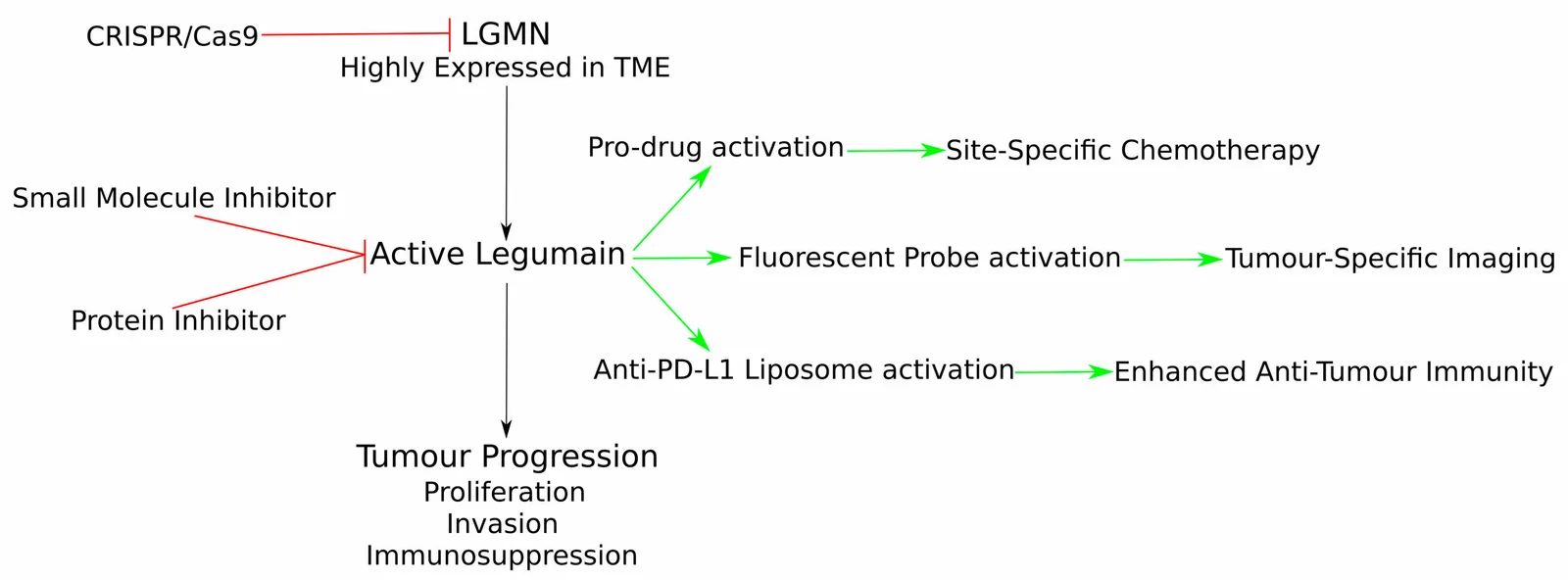
Therapeutic Strategies Targeting and Exploiting Legumain in Oncology. Two distinct therapeutic philosophies have been developed. (Red) Legumain as the Target: This strategy aims to directly inhibit legumain’s pro-tumoural activity using small molecules or protein inhibitors or eliminate its production via gene editing. (Green) Legumain as the Tool: This strategy exploits the high, localised legumain activity in the tumour microenvironment as a specific trigger to activate otherwise inert prodrugs, diagnostic probes, or advanced immunotherapies, thereby achieving tumour-specific effects and minimising systemic toxicity.

The comprehensive evidence from molecular, preclinical, and clinical studies has established legumain as a highly attractive molecule for cancer intervention. This has led to the development of two distinct but complementary therapeutic philosophies. The first strategy treats legumain as the direct therapeutic target, aiming to inhibit its pro-tumoural functions. The second strategy leverages its unique, localised activity in the tumour microenvironment, using legumain as a precision tool to activate drugs and imaging agents specifically at the disease site (Figure 3).

It is important to note that these approaches differ substantially in translational maturity. To date, no legumain-targeted agent has entered human clinical trials specifically for cancer or cardiovascular indications; esomeprazole is a notable exception in that it is an FDA-approved drug for an unrelated indication (gastric acid suppression) that has been *repurposed* and tested only in preclinical legumain-inhibition models [36]. All other strategies discussed below—including natural-product inhibitors, CRISPR/Cas9 gene editing, chimeric protein inhibitors, activity-based imaging probes, and legumain-activatable prodrugs/nanomedicines—remain at the in vitro or animal-model stage. Among these, small-molecule and prodrug/nanomedicine approaches generally have the most robust in vivo (animal-model) support, whereas gene-editing and several imaging-probe strategies currently remain largely at the proof-of-concept, cell-based stage.

This approach is founded on the principle that blocking legumain’s function will directly impede cancer progression. A multi-pronged effort is underway to develop potent legumain inhibitors. This includes leveraging endogenous protein inhibitors like cystatin E/M, which can be internalised by melanoma cells to reduce their malignant phenotype [56]. A range of small molecules have been identified or developed, including the repurposed FDA-approved drug esomeprazole [36], potent xanthine derivatives [27], and natural product extracts [37], all of which have shown efficacy in suppressing metastasis in preclinical breast cancer models. Protein engineering has also yielded novel chimeric inhibitors, such as a modified oryzacystatin-I, which reduces the viability of colorectal cancer cells [51]. At the genomic level, strategies aiming to eliminate legumain entirely, such as CRISPR/Cas9-mediated gene editing, have successfully impaired the metastatic capacity of breast cancer cells in vivo, representing a potential future therapeutic avenue [38].

This second strategy exploits the high, localised expression of active legumain in the TME as a specific biomarker for activating theranostic agents. Here, legumain’s enzymatic activity is not the problem to be solved but the key to unlocking a solution.

For diagnostics, this has led to the creation of “turn-on” fluorescent probes. These probes are engineered with a quencher molecule that is cleaved off by legumain, causing them to light up and enabling real-time imaging of their activity in living cells and tumours [63,64]. This concept has been extended to clinical imaging modalities like PET, with the development of radiolabelled tracers ([18F]SF-AAN) that accumulate specifically in legumain-positive tumours [65], though challenges with metabolic stability remain for some probe designs [66,67].

For therapeutics, this “smart” activation mechanism is used to design sophisticated drug delivery systems. These include legumain-cleavable prodrugs of potent chemotherapeutics like doxorubicin, which remain inert in circulation but become toxic upon activation by legumain in the tumour [68]. This approach has been integrated into advanced nanomedicines, such as dual-responsive nanoparticles that release their payload in response to both legumain and the acidic TME [69]. This strategy has been powerfully combined with immunotherapy, using legumain-activatable systems to deliver anti-PD-L1 antibodies [52] or immunogenic cell death agents like oxaliplatin [70] to enhance anti-tumour immunity. Further innovations include bioengineered macrophages [71] and nanoparticles designed to specifically target TAMs [72], expanding the toolkit of legumain-responsive drug-delivery platforms beyond conventional prodrug design, all using legumain as the specific biochemical trigger to unleash their therapeutic potential at the tumour site.

In summary, legumain emerges from the literature not merely as a bystander but as an active contributor to cancer pathogenesis. The evidence compellingly demonstrates its multifaceted involvement, beginning with its dysregulation at the molecular level through complex genetic and epigenetic networks. This aberrant expression translates directly into enhanced tumour aggression, driving the core hallmarks of cancer, including proliferation, invasion, and metastasis across a wide array of malignancies. Furthermore, legumain’s influence extends beyond the cancer cell itself, as it actively sculpts an immunosuppressive tumour microenvironment by modulating the function of key immune cells like macrophages. This body of work, spanning from fundamental cell biology to preclinical and extensive human studies, firmly establishes legumain as a robust biomarker of poor prognosis and therapy resistance. Consequently, it represents an extensively studied and versatile subject for cancer intervention, though largely at the preclinical stage. The development of strategies that both directly target its enzymatic activity and cleverly exploit it as a tumour-specific trigger heralds a promising new frontier in the ongoing effort to develop more precise and effective cancer therapies.

## 3. The Role of Legumain in Cardiovascular Disease

In parallel with its established role in cancer, legumain is emerging as a significant contributor to the pathophysiology of cardiovascular diseases (CVDs). Its expression is upregulated in atherosclerotic plaques, and it actively participates in vascular inflammation, plaque instability, and adverse cardiac remodelling. Following a logical progression, this section will first detail the mechanistic actions of legumain as elucidated in preclinical models and then discuss its clinical relevance and potential as a biomarker in human cardiovascular conditions.

### 3.1. Mechanistic Roles of Legumain in Preclinical Models of CVD

In vitro and in vivo animal studies have been crucial in moving legumain beyond a simple association with CVD to establishing it as a causal factor in disease progression. These models have revealed its direct impact on key cellular players in atherosclerosis, hypertension, and cardiac remodelling (Table 3).

**Figure 4:**
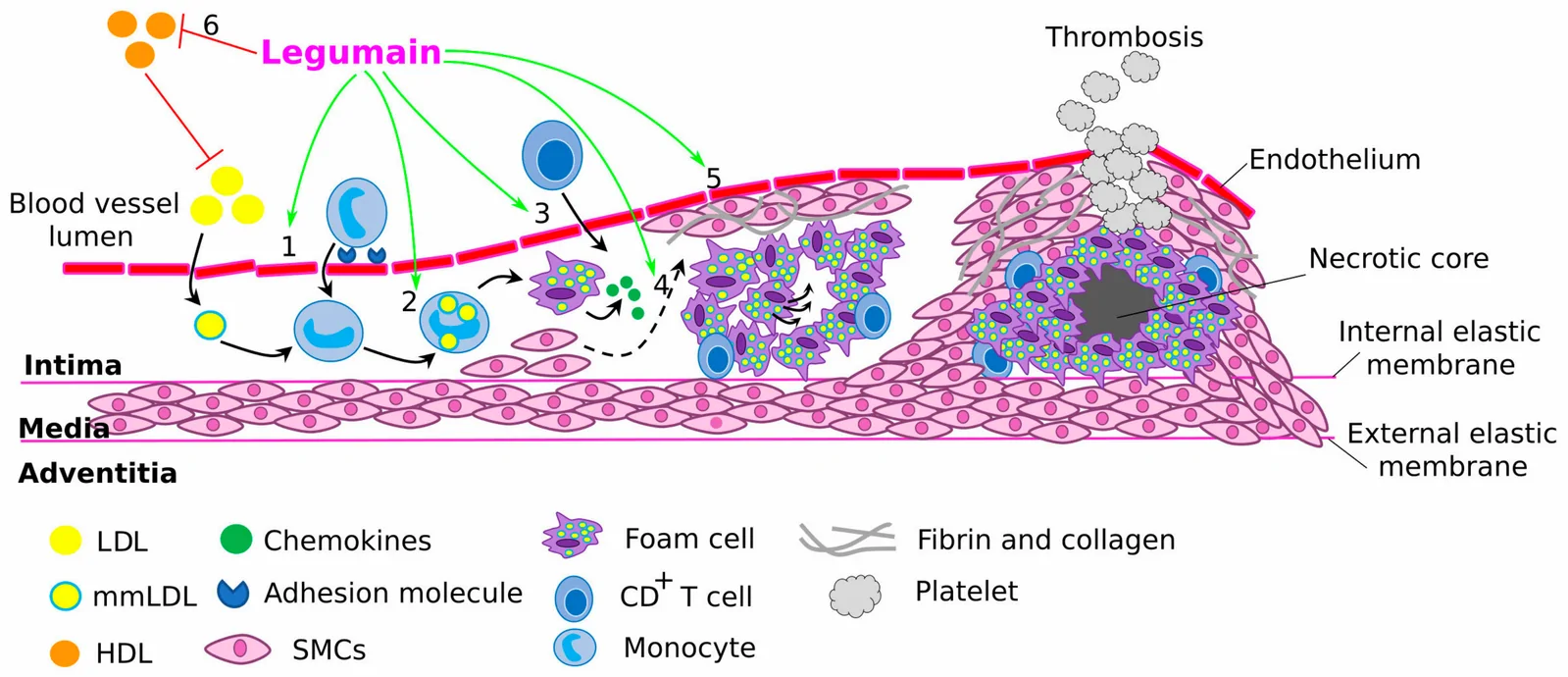
The Pro-Atherogenic Mechanisms of Legumain in the Vascular Wall. Legumain contributes to multiple stages of atherosclerotic plaque development. It promotes a pro-inflammatory macrophage phenotype, enhances their survival and transformation into foam cells, and increases accumulation of CD4+ T cells in plaque. Legumain also stimulates the migration of smooth muscle cells (SMCs) and their production of extracellular matrix, contributing to the growth of the plaque. At a molecular level, a key mechanism is the cleavage of APOA1, which disrupts HDL-mediated cholesterol efflux and accelerates lipid accumulation. (1) Promotes monocyte infiltration; (2) promotes pro-inflammatory (M1) macrophage phenotype switch and foam cell formation and enhances survival via autophagy; (3) enhances intraplaque T cell accumulation by improving their function and survival; (4) promotes SMC migration; (5) increases ECM production; (6) impairs HDL function and cholesterol efflux. Green arrows represent activation; red blunt lines represent inhibition.

Preclinical models provide compelling evidence for legumain’s direct role in the formation and progression of atherosclerotic plaques. In *Apoe*^−/−^ mice, chronic infusion of legumain potentiated the development of lesions, which were characterised by increased macrophage and smooth muscle cell accumulation and collagen deposition [73]. Mechanistically, legumain enhances the pro-inflammatory M1 phenotype in macrophages, promotes oxidised LDL-induced foam cell formation, and regulates macrophage survival by enhancing autophagy (Figure 4) [73,74]. In a rat model, legumain expression in plaques correlated linearly with the Smad3 pathway, with serum levels approximately doubling in atherosclerotic animals (~16 µg/L) compared to controls (~8 µg/L) [77].

A key molecular function appears to be the cleavage of apolipoprotein A1 (APOA1), which impairs HDL-mediated cholesterol efflux and accelerates atherogenesis; genetic deletion or pharmacological inhibition of legumain substantially diminished atherosclerosis in mouse models [75]. Furthermore, legumain influences the immune response within plaques, as its deletion in *Apoe*^−/−^ mice reduced CD4+ T cell accumulation by impairing their survival and function [76].

Legumain’s pathological influence extends beyond atherosclerosis. In mouse models of hypertension, inhibition of legumain prevented angiotensin II-induced hypertension by preserving the function of protective regulatory T cells (Tregs) [78]. In the context of acute myocardial infarction (AMI), blockade of legumain in mice improved cardiac function and attenuated adverse remodelling, partly by reducing MMP-2 activation [79]. Complementing these findings, proteomic studies have identified novel potential substrates, including the mitochondrial protein frataxin, implicating legumain in cellular metabolism [81].

### 3.2. Legumain as a Clinical Biomarker in Human Cardiovascular Disease

The functional roles established in preclinical models are strongly supported by clinical data, which highlight circulating legumain as a promising, albeit complex, biomarker for various CVDs (Table 4).

A consistent finding is the association of elevated legumain with atherosclerosis. Patients with carotid stenosis have significantly higher plasma legumain levels (median 2.0 ng/mL) compared to healthy controls (1.5 ng/mL), with expression being highest in symptomatic plaques [82]. Another study found even higher levels in atherosclerotic patients (3.52 ng/mL) versus controls (1.56 ng/mL), establishing it as a diagnostic and prognostic marker [83].

Legumain appears specifically linked to plaque vulnerability. In patients with coronary artery disease (CAD), elevated plasma legumain was associated with complex, unstable lesions, with a proposed risk cut-off of >5.5 ng/mL [84]. Furthermore, levels were markedly elevated in patients with unstable angina (median 25.9 ng/mL) compared to stable angina (14.8 ng/mL) and controls (8.6 ng/mL), correlating with established high-risk plaque features such as a thin fibrous cap, a large necrotic core, and positive (expansive) vascular remodelling [85]. This association extends to high-risk populations, including patients with peripheral artery disease (PAD), who show higher serum concentrations (median 11.9 µg/L in PAD vs. 7.6 µg/L in non-PAD patients) [86]. It is also elevated in adults with familial hypercholesterolemia (FH) on statin therapy (median 4.9 pg/mL vs. 3.3 pg/mL in controls) [87]. The wide variation in absolute concentrations reported (ranging from pg/mL to µg/L) likely reflects differences in assay methodologies and patient cohorts but consistently points to a relative increase in legumain during atherosclerotic disease.

The role of legumain during acute events is more complex. In patients with AMI, higher plasma legumain on admission (median 5.9 µg/L vs. 4.4 µg/L in controls) was a significant predictor of long-term all-cause mortality [79]. Similarly, in a large cohort of patients with acute coronary syndrome (ACS), risk of adverse events increased across legumain concentration quartiles (ranging from <1.97 ng/mL to >3.86 ng/mL), although this was not independent of other established biomarkers [88]. This finding is an important reminder that demonstrating an association between circulating legumain and disease severity or outcome is not equivalent to demonstrating incremental diagnostic or prognostic value over established clinical markers—a distinction that should be borne in mind when interpreting the studies summarised in Table 4.

Intriguingly, evidence also points towards a potentially protective role. In a population of patients with ST-segment elevation myocardial infarction (STEMI), high circulating legumain was paradoxically associated with an *improved* outcome [80]. This study also demonstrated that legumain is released by activated platelets, suggesting a complex involvement in the acute thrombotic and inflammatory response that warrants further investigation.

In conclusion, the evidence presented indicates that legumain is a significant, context-dependent contributor in the pathogenesis of cardiovascular disease. Preclinical studies have moved its role beyond mere association, demonstrating that legumain actively promotes the core processes of atherosclerosis by driving vascular inflammation, impairing cholesterol metabolism, and contributing to adverse immune responses within the plaque. This pro-atherogenic function is further compounded by its detrimental effects on cardiac remodelling following myocardial infarction and its contribution to the development of hypertension. In the clinical arena, circulating legumain has emerged as a promising, though complex, biomarker associated with plaque presence, disease severity, and features of plaque vulnerability (e.g., thin-cap fibroatheroma, necrotic core expansion, and positive remodelling) across various patient populations. While its precise prognostic role during acute coronary events requires further elucidation, the collective data supports a role for legumain as a context-dependent contributor to chronic cardiovascular pathology, marking it as a compelling novel target for future diagnostic and therapeutic strategies aimed at mitigating the burden of these widespread diseases.

## 4. Synthesis, Unresolved Questions, and Future Directions

The preceding sections have established legumain as a significant, context-dependent mediator in the distinct pathologies of cancer and cardiovascular disease. While these fields are often studied in isolation, a synthesis of the literature reveals remarkable parallels in legumain’s function, common signalling hubs it modulates, and shared unanswered questions. This section will critically evaluate these convergences and controversies, address the current limitations in the field, and propose key directions for future research that could benefit both domains.

### 4.1. A Common Mechanistic Blueprint: Inflammation, Remodelling, and Immune Modulation

It should be noted that the M1/M2 classification, while a useful heuristic for describing the pro-inflammatory versus immunosuppressive/tissue-remodelling extremes of macrophage phenotype, is now recognised as an oversimplification of a continuous and highly heterogeneous spectrum of macrophage activation states that varies with tissue context, ontogeny, and disease stage. The available legumain literature has largely been framed within this binary model, and future studies employing single-cell transcriptomic or proteomic approaches will be needed to resolve which specific macrophage subpopulations and activation states are influenced by legumain in the tumour microenvironment and the atherosclerotic plaque.

**Figure 5:**
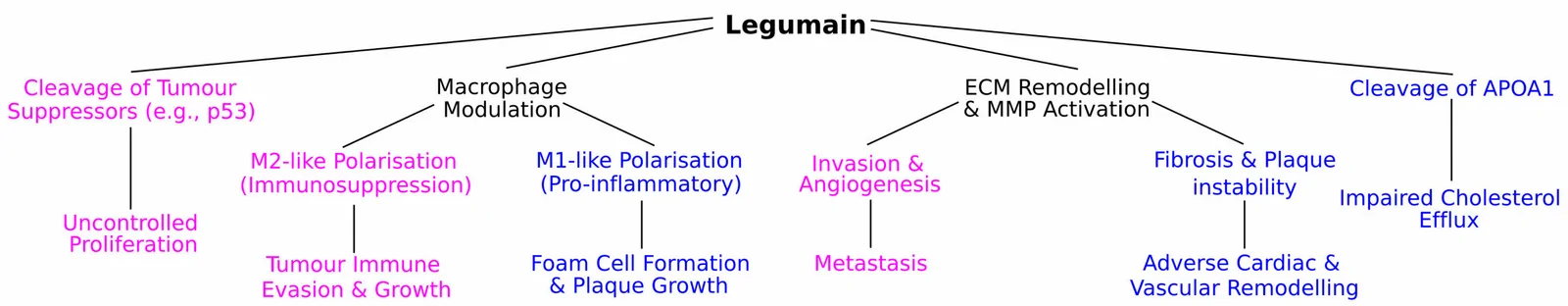
Convergent Mechanisms of Legumain in Cancer (magenta) and Cardiovascular Disease (blue). Legumain employs a common mechanistic toolkit in both pathologies, centrally involving macrophage modulation and extracellular matrix remodelling. However, these shared mechanisms lead to distinct, disease-specific outcomes such as immunosuppression and metastasis in cancer versus inflammation and plaque formation in CVD.

A striking convergence between cancer and CVD is legumain’s role as a key contributor to tissue remodelling and localised inflammation. In both contexts, legumain is not merely a bystander but an active participant in altering the microenvironment to favour disease progression (Figure 5). Its ability to cleave and activate matrix metalloproteinases (MMPs), such as MMP-2, is a shared mechanism that promotes tumour invasion in breast cancer and drives adverse cardiac remodelling post-myocardial infarction. Similarly, its capacity to degrade extracellular matrix components like fibronectin is fundamental to both cancer cell metastasis and the structural changes seen in atherosclerotic plaques.

Perhaps the most significant parallel is legumain’s profound influence on macrophages. In both glioblastoma and gastric cancer, tumour-secreted legumain polarises macrophages towards an immunosuppressive, pro-tumoural M2 phenotype. This is remarkably similar to its function in atherosclerosis, where it promotes a pro-inflammatory M1 macrophage phenotype that drives plaque formation. While the resulting macrophage phenotype (M1 vs. M2) appears context-dependent, the underlying principle is the same: legumain is a key paracrine signal that hijacks innate immune cells to create a disease-permissive niche. This shared reliance on macrophage modulation highlights a common axis of inflammation that is central to both chronic diseases.

### 4.2. Controversies and Context-Dependent Functions

**Figure 6:**
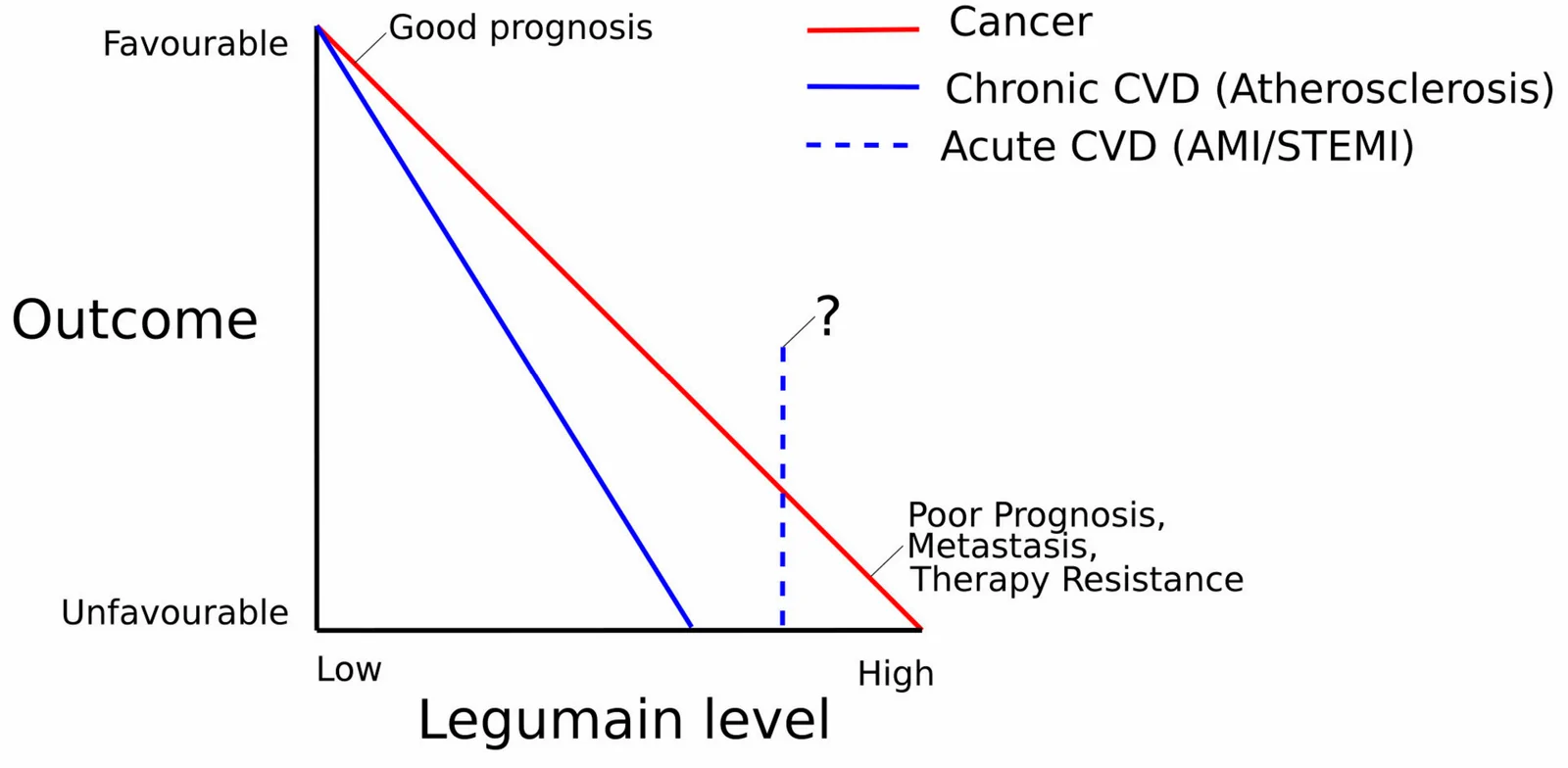
Divergent Clinical Roles of Legumain in Cancer and Cardiovascular Disease. As a clinical biomarker, the role of legumain diverges. In most cancers, elevated legumain is unambiguously associated with poor prognosis. In cardiovascular disease, its role is more complex: while high levels are linked to poor outcomes in chronic atherosclerosis, they have been paradoxically associated with favourable outcomes in acute events like STEMI, highlighting a key area of controversy and future investigation.

Despite these parallels, the literature is not without its inconsistencies, which underscore the context-dependent nature of legumain’s function. The most prominent controversy is its seemingly dual role in acute cardiovascular events (Figure 6). While chronically elevated legumain is consistently associated with worse outcomes in atherosclerosis, high levels in the acute phase of a STEMI have been linked to *improved* survival. This paradox may be explained by its release from activated platelets during acute thrombosis, suggesting a potential role in haemostasis or early inflammatory resolution that is distinct from its chronic, pro-fibrotic functions.

This apparent paradox likely reflects several non-mutually exclusive factors that remain to be formally dissected. First, the acute versus chronic phase of disease may involve distinct biological roles: chronically elevated legumain may reflect sustained pro-inflammatory and pro-fibrotic activity within the vessel wall, whereas an acute rise during STEMI may instead reflect a transient release event associated with platelet activation and clot formation. Second, the cellular source of circulating legumain likely differs between these settings—plaque macrophages and smooth muscle cells in chronic atherosclerosis versus activated platelets in acute thrombosis [80]—and legumain originating from different cell types may carry different post-translational modifications or activity states. Third, most clinical assays measure total immunoreactive legumain rather than distinguishing circulating versus tissue-bound, or pro- versus active, enzymatic forms (see Section 4.3), so an identical plasma concentration could represent biologically distinct pools of the protein in the acute and chronic settings. Disentangling these possibilities will require studies that combine temporal sampling, cell-of-origin tracing, and activity-based assays.

Similarly, in the tumour microenvironment, while legumain typically promotes an immunosuppressive state, studies in breast cancer have shown that its deletion in macrophages can paradoxically induce tumour cell senescence via IL-1β signalling [34]. This suggests that the ultimate outcome of legumain’s activity is finely tuned by the specific cytokine milieu, the presence of other cell types, and the stage of the disease, cautioning against a “one-size-fits-all” view of its function.

### 4.3. Current Limitations and Methodological Challenges

To advance the field, several key limitations must be addressed. A major challenge, evident from the clinical data, is the lack of standardised assays for measuring legumain concentration. Reported levels in patient plasma span an enormous range, from pg/mL to µg/L, making cross-study comparisons nearly impossible. This discrepancy likely arises from differences in antibody specificity (detecting pro- vs. active forms), assay platforms (ELISA vs. mass spectrometry), and sample handling. Establishing a standardised, well-characterised assay is a critical and immediate need before legumain can be implemented as a validated clinical biomarker.

A related and equally important limitation is that most clinical studies quantify total circulating legumain immunoreactivity (typically by ELISA) rather than its enzymatic activity. Because legumain circulates in multiple molecular forms—the inactive 56 kDa zymogen, partially processed intermediates, and the fully active mature protease [4,5]—antibody-based assays may not distinguish between biologically inert and catalytically active pools. Two patients with identical total legumain concentrations could therefore have markedly different levels of active enzyme. Activity-based probes or assays capable of specifically detecting the mature, catalytically competent form are needed to determine whether circulating concentration, enzymatic activity, or both carry independent prognostic or mechanistic significance.

Furthermore, while many studies have focused on legumain’s proteolytic AEP activity, its other functions—such as its peptide ligase (PAL) activity or its non-proteolytic signalling role via integrin binding—are less understood in the context of disease. It is plausible that these different functions contribute to its context-dependent effects, and a lack of tools to specifically measure or inhibit each activity in vivo currently limits our understanding.

### 4.4. Future Directions: Bridging the Gaps

Building on this synthesis, several avenues of research promise to resolve these outstanding questions and translate our knowledge into clinical benefit.

1. Elucidating the “Functional Switch”: Future research must focus on what determines legumain’s functional output. What molecular signals dictate whether it acts as a protease or a ligase? How does the cellular microenvironment (e.g., pH, redox state, and co-factors) determine whether its influence on macrophages is pro-inflammatory or immunosuppressive? Studies using single-cell spatial transcriptomics in diseased tissues could map legumain expression and its functional state with cellular context.
2. Developing Function-Specific Inhibitors: There is a clear need for therapeutic tools that can distinguish between legumain’s different activities. Developing small molecules or biologics that can selectively block its AEP activity without affecting its integrin-binding or ligase functions would be a major breakthrough, allowing researchers to dissect its roles with greater precision and potentially leading to more targeted therapies with fewer off-target effects.
3. A further consideration for translation is that legumain performs essential physiological functions, including antigen processing in the endolysosomal pathway and normal protein turnover in the kidney, liver, and spleen [3]. Systemic pharmacological inhibition may therefore carry on-target risks that are not captured by disease-specific preclinical models. This concern is compounded by the apparently protective association of acutely elevated legumain in STEMI [80] (Section 4.2): an inhibitor developed to counter its chronic, pro-atherogenic effects could, in principle, be detrimental if administered during an acute cardiovascular event. Future preclinical safety studies should therefore evaluate legumain inhibitors across both chronic and acute disease contexts, and ideally in a tissue- or context-restricted manner rather than through systemic blockade.
4. Connecting the Fields—The Cardio-Oncology Axis: Legumain stands at the nexus of cardio-oncology. Many cancer therapies are cardiotoxic, and chronic inflammation drives both diseases. Is legumain a common mediator in these processes? Future studies should investigate whether legumain levels predict cardiotoxicity in cancer patients undergoing treatment, or if cancer patients with high legumain are at greater risk of developing cardiovascular disease post-treatment.
5. Standardisation and Validation of Clinical Assays: A concerted, multi-centre effort is required to develop and validate a reference standard for legumain measurement. This would harmonise data across studies and is a prerequisite for its translation into a routine diagnostic or prognostic tool for either cancer or CVD.
6. **Distinguishing Circulating Concentration from Enzymatic Activity:** A key unresolved question is whether the clinical associations reported throughout Section 2.3 and Section 3.2 reflect legumain’s active, catalytically competent form or simply its total immunoreactive abundance. Prospective studies incorporating activity-based probes alongside conventional immunoassays are needed to determine whether concentration, activity, or both carry independent prognostic value.

## 5. Conclusions: Legumain as a Context-Dependent Mediator in Chronic Disease

The journey through the diverse literature on legumain reveals a molecule of remarkable complexity and profound consequence. It is clear that viewing legumain as a simple protease is insufficient; it functions as a sophisticated biological rheostat, capable of fine-tuning inflammation, immunity, and tissue architecture through its multiple enzymatic and signalling capacities. Its ability to act as an endopeptidase, a carboxypeptidase, a ligase, and a non-proteolytic signalling partner grants it a level of functional plasticity that positions it as a significant, context-dependent contributor to the progression of both cancer and cardiovascular disease.

Upon synthesising the evidence, a common pathological axis emerges. In both malignancies and atherosclerosis, legumain’s primary role is to sculpt the local microenvironment into a state that permits and promotes disease. It frequently acts as a key regulator of the cellular microenvironment, although its precise role is highly context-dependent, directing the behaviour of immune cells—most notably macrophages—and orchestrating the remodelling of the extracellular matrix. Whether it is creating an immunosuppressive niche for a growing tumour or a pro-inflammatory, fibrotic environment for an unstable plaque, the underlying principle remains the same: legumain dysregulation represents a fundamental breakdown in local tissue homeostasis. This convergence suggests that the mechanisms driving chronic inflammation and tissue disruption are deeply shared across these seemingly disparate diseases.

This deep mechanistic understanding has, in turn, inspired a sophisticated, dual-pronged therapeutic paradigm. The field is not only pursuing strategies to inhibit legumain’s detrimental activities but is also cleverly harnessing its localised enzymatic power as a precision tool to activate tumour-specific drugs and imaging agents. This parallel development of legumain as both a “target” and a “trigger” is a testament to the ingenuity of the field and highlights its immense potential in the era of personalised medicine. It offers the rare opportunity to either silence a key driver of disease or, conversely, to use its presence as a key to unlock a tailored therapeutic response.

However, the path to clinical translation requires a concerted effort to address the field’s current limitations. Methodological inconsistencies in measuring legumain levels—together with context-dependent and, in some settings, opposing associations with outcome (e.g., the divergent findings in chronic atherosclerosis versus acute STEMI, or the lack of prognostic value in BCP-ALL)—indicate that legumain is unlikely to function as a single, disease-agnostic biomarker. Its clinical utility will more plausibly be realised as a context- and disease-specific biomarker, requiring separate validation within each clinical setting. Furthermore, our understanding of how the cellular context dictates legumain’s functional switches—from protease to ligase, from pro-inflammatory to immunosuppressive—remains in its infancy. Future research must therefore focus on developing standardised assays and function-specific molecular probes to dissect these complex roles with greater clarity.

Ultimately, legumain stands at the critical intersection of pathology and therapeutic opportunity. It offers a unique window into the shared mechanisms of chronic disease and serves as a powerful reminder that a single molecule can have a vast and varied impact. By continuing to unravel its complexities and bridging the research between fields like oncology and cardiology, we can fully unlock its potential. The challenge ahead is to translate our rich understanding of legumain from a compelling biological narrative into a spectrum of innovative therapies that can profoundly impact a wide range of human diseases.

Rather than positioning legumain as a universal master regulator, we suggest that its most robust and generalisable feature across cancer and cardiovascular disease is its context-dependent functional plasticity—the fact that its pathological impact depends critically on cell type, subcellular localisation, disease stage, and local microenvironmental cues. We consider this context-dependency, rather than a singular unifying mechanism, to be the most important conclusion to emerge from this synthesis.

## Figures and Tables

**Table 1:** Preclinical Evidence for Legumain in Cancer.

Cancer Type	Preclinical Model/System	Key Mechanistic Finding	Reference
Breast Cancer	Mouse metastasis models; cell culture	AEP blockade inhibits metastasis; promotes migration via integrin αvβ3; suppression attenuates bone pain in metastasis models	[27,28,29]
Breast Cancer	Polyomavirus middle-T antigen transgenic mouse mammary tumour model	AEP suppression inhibits tumour formation and metastasis	[30]
Breast Cancer	Mouse lung-metastasis model; cell culture	MIF inhibitor + AEP inhibitor combination reduces lung metastasis; AEP regulates lysosome homeostasis via phosphoinositide composition	[31,32]
Breast Cancer	Cell culture (USP17 deubiquitination mechanism)	USP17 deubiquitinates legumain, suppressing tumorigenesis via ERK inactivation	[33]
Breast Cancer	Mouse tumour model with macrophage-specific legumain deletion	Legumain-deficient macrophages induce tumour cell senescence via JAK1/STAT1	[34]
Breast Cancer	Mouse metastasis model	C/EBPβ/AEP signalling mediates oxidative stress and metastasis	[35]
Breast Cancer	Mouse metastasis model; in vitro	Esomeprazole and natural-product extracts inhibit legumain and suppress metastasis	[36,37]
Breast Cancer	Mouse model; CRISPR/Cas9 LGMN editing	Cas9-mediated LGMN editing represses metastatic capacity	[38]
Glioblastoma	Xenograft model	Legumain cleaves Tmod3, promoting migration/proliferation; inactivates WT-p53	[39,40]
Glioblastoma	Glioblastoma cell lines/xenograft models	LGMNP1 pseudogene and circLGMN act as ceRNA sponges (miR-495-3p, miR-127-3p) to upregulate legumain	[41,42]
Glioblastoma	Syngeneic mouse GBM models	CLOCK–BMAL1–HIF1α axis and hypoxia drive legumain-dependent TAM polarisation and immunosuppression	[43,44]
Glioblastoma	Preclinical GBM models	Targeting legumain-mediated cell–cell interaction sensitises GBM to immunotherapy	[45]
Gastric Cancer	In vitro and in vivo (mouse xenograft)	TAM-derived legumain promotes proliferation, angiogenesis, and invasion	[46]
Gastric Cancer	Cell culture; metastatic tissue specimens	PCBP1/miR-3978 complex disruption de-represses legumain during metastasis	[47]
Gastric Cancer	Cell culture; *H. pylori* infection model	Oxidation of Cys219 inhibits intracellular processing, enhancing pro-enzyme secretion	[48]
Colorectal Cancer	Cell line study	AEP promotes MEK/ERK phosphorylation, contributing to cetuximab resistance	[49]
Colorectal Cancer	Murine colitis-to-cancer model (N-terminomics)	Identifies candidate legumain substrates in the inflammation-to-cancer transition	[50]
Colorectal Cancer	In vitro inhibitor assay; mouse colon-cancer model	Chimeric oryzacystatin-I inhibitor reduces cell viability; legumain-cleavable liposomes deliver anti-PD-L1/doxorubicin	[51,52]
Ovarian Cancer	Subcutaneous mouse xenograft (EOC)	Legumain overexpression enhances tumour growth and microvessel density	[53]
Thyroid Carcinoma	Cell culture (autophagy pathway)	Pseudogene-driven legumain promotes proliferation/invasion via miR-495/autophagy axis	[54]
Pancreatic Cancer	Ex vivo dendritic-cell/exosome co-culture	Cancer-derived exosomes upregulate legumain in dendritic cells	[55]
Melanoma	Human melanoma cell lines	Internalised cystatin E/M inhibits legumain activity and reduces migration/invasion	[56]
Choriocarcinoma	Xenograft model	Legumain overexpression reverses the anti-proliferative effect of metformin	[57]
OSCC	Mouse model of cancer-associated pain	Legumain activates PAR2 on trigeminal neurons to cause pain	[58]

**Table 2:** Clinical Evidence for Legumain in Cancer.

Cancer Type	Patient Cohort/Sample Type	Clinical Finding	Reference
Breast Cancer (DCIS)	Ductal carcinoma in situ patient cohort	Independent predictor of invasive recurrence	[59]
Breast Cancer	Patient tumour samples	High legumain associated with radiotherapy resistance and suppressed DNA-damage response	[60]
Glioblastoma	Patient plasma	Levels correlate with tumour size, decrease after resection	[40]
Gastric Cancer	Patient tumour samples	Independent prognostic factor; associated with diffuse-type GC and peritoneal metastasis	[61]
Colorectal Cancer	Metastatic CRC patients on cetuximab	High expression associated with shorter progression-free survival	[49]
Ovarian Cancer (EOC)	Patient tissue/ascites; exosomes	Exosomal AEP/ITGA5B1 complex predicts poor survival, mediates metastasis	[19]
Thyroid Carcinoma	Patient tumour samples	High expression correlates with lower overall survival	[54]
BCP-ALL	Paediatric patient cohort	Overexpressed in some genetic subtypes; not associated with relapse-free survival	[62]

*Abbreviations*: AEP: Asparaginyl Endopeptidase; AKT: Protein Kinase B; BCP-ALL: B-Cell Precursor Acute Lymphoblastic Leukaemia; BMAL1: Brain and Muscle ARNT-Like 1; C/EBPβ: CCAAT-Enhancer-Binding Protein Beta; ceRNA: Competing Endogenous RNA; circLGMN: Circular RNA Legumain; CLOCK: Circadian Locomotor Output Cycles Kaput; ERK: Extracellular Signal-Regulated Kinase; GC: Gastric Cancer; GSK3β: Glycogen Synthase Kinase 3 Beta; HIF1α: Hypoxia-Inducible Factor 1-Alpha; ITGA5B1: Integrin Subunit Alpha 5 Beta 1; LGMNP1: Legumain Pseudogene 1; MEK: Mitogen-Activated Protein Kinase; miR: microRNA; MMP-2: Matrix Metalloproteinase-2; OSCC: Oral Squamous Cell Carcinoma; PAR2: Protease-Activated Receptor 2; PCBP1: Poly(rC)-Binding Protein 1; PD-1: Programmed Cell Death Protein 1; PI3K: Phosphoinositide 3-Kinase; p53: Tumour Protein p53; STAT3: Signal Transducer and Activator of Transcription 3; TAM: Tumour-Associated Macrophage; TME: Tumour Microenvironment; Tmod3: Tropomodulin-3; USP17: Ubiquitin-Specific Peptidase 17; WT: Wild-Type.

**Table 3:** Preclinical Evidence for Legumain in Cardiovascular Disease.

Disease Context	Model	Key Finding	Reference
Atherosclerosis	Apoe^−^/^−^ mice	Chronic legumain infusion potentiates lesion development, vascular remodelling, macrophage/SMC accumulation, collagen deposition	[73]
Atherosclerosis	Macrophage cell culture	Legumain protects macrophages from oxLDL-induced apoptosis via autophagy	[74]
Atherosclerosis	Apoe^−^/^−^ and Ldlr^−^/^−^ mice	Legumain cleaves APOA1, impairing cholesterol efflux/HDL formation, accelerating atherosclerosis	[75]
Atherosclerosis	Lgmn^−^/^−^Apoe^−^/^−^ mice	Legumain deletion attenuates atherosclerosis by impairing CD4^+^ memory T-cell survival/function	[76]
Atherosclerosis	Rat model	Plaque legumain correlates with Smad3 pathway; attenuated by statins (serum ~16 vs. ~8 µg/L)	[77]
Hypertension	Mouse model (Ang II-induced)	Legumain inhibition prevents hypertension by preserving Treg function via TRAF6	[78]
Acute MI	Mouse model	Pharmacological legumain blockade improves cardiac function post-AMI	[79]
Acute events	In vitro (activated platelets)	Legumain is released by activated platelets	[80]
General mechanistic	Macrophage cell culture (proteomics)	Identifies frataxin as a novel substrate, implicating legumain in mitochondrial/iron metabolism	[81]

**Table 4:** Clinical Evidence for Legumain in Cardiovascular Disease.

Disease Context	Population	Reported Legumain Levels	Clinical Finding	Reference
Carotid atherosclerosis	Human patients	Plasma: 2.0 vs. 1.5 ng/mL	Elevated in carotid stenosis, highest in symptomatic plaques	[82]
Atherosclerosis	Human patients	Plasma: 3.52 vs. 1.56 ng/mL	Diagnostic/prognostic marker correlating with severity	[83]
CAD	Human patients	Cut-off > 5.5 ng/mL	Associated with complex, unstable coronary lesions	[84]
Angina	Human patients	Plasma (median): UA 25.9; SA 14.8; control 8.6 ng/mL	Correlates with high-risk plaque features (thin fibrous cap, large necrotic core, positive remodelling)	[85]
PAD in T2DM	Human patients	Serum (median): PAD 11.9 vs. no-PAD 7.6 µg/L	Independently associated with PAD risk	[86]
Familial hypercholesterolemia	Human patients on statins	Plasma (median): 4.9 vs. 3.3 pg/mL	Elevated in FH patients on statin therapy	[87]
Hypertension	Human patients	mRNA elevated in CD4^+^ T cells	Correlates with hypertensive status	[78]
Acute MI	Human patients	Plasma (median): 5.9 vs. 4.4 µg/L	Predicts all-cause mortality post-AMI	[79]
ACS (PLATO substudy)	Human patients	Quartiles: <1.97 to >3.86 ng/mL	Associated with adverse events, not independent of established biomarkers	[88]
STEMI	Human patients	Not specified	Paradoxically associated with improved outcome	[80]

*N/A: Not Applicable or Not Available in the provided source material. Note the different units used across studies *(ng/mL, µg/L, pg/mL), *which is an important point for discussion in the main text*.

## Data Availability

No new data were created or analyzed in this study. Data sharing is not applicable to this article.
